# Delphinidins from Maqui Berry (*Aristotelia chilensis*) ameliorate the subcellular organelle damage induced by blue light exposure in murine photoreceptor-derived cells

**DOI:** 10.1186/s12906-023-04322-z

**Published:** 2024-01-02

**Authors:** Kanta Yamazaki, Kodai Ishida, Wataru Otsu, Aomi Muramatsu, Shinsuke Nakamura, Wakana Yamada, Hideshi Tsusaki, Hiroshi Shimoda, Hideaki Hara, Masamitsu Shimazawa

**Affiliations:** 1 Molecular Pharmacology, Department of Biofunctional Evaluation, Gifu Pharmaceutical University, 1-25-4 Daigaku-nishi, Gifu, 501-1196 Japan; 2 Department of Biomedical Research Laboratory, Gifu Pharmaceutical University, 1-25-4 Daigaku-nishi, Gifu, 501-1196 Japan; 3 Research & Development Division, Oryza Oil & Fat Chemical Co., Ltd, 1 Numata, Kitagata- cho, Ichinomiya, Aichi 493-8001 Japan

**Keywords:** Anthocyanin, Integrated stress response, Light-emitting diode, Mitochondria, Oxidative stress, Photoreceptor

## Abstract

**Background:**

Blue light exposure is known to induce reactive oxygen species (ROS) production and increased endoplasmic reticulum stress, leading to apoptosis of photoreceptors. Maqui berry (*Aristotelia chilensis*) is a fruit enriched in anthocyanins, known for beneficial biological activities such as antioxidation. In this study, we investigated the effects of Maqui berry extract (MBE) and its constituents on the subcellular damage induced by blue light irradiation in mouse retina-derived 661W cells.

**Methods:**

We evaluated the effects of MBE and its main delphinidins, delphinidin 3-O-sambubioside-5-O-glucoside (D3S5G) and delphinidin 3,5-O-diglucoside (D3G5G), on blue light-induced damage on retinal cell line 661W cells. We investigated cell death, the production of ROS, and changes in organelle morphology using fluorescence microscopy. The signaling pathway linked to stress response was evaluated by immunoblotting in the whole cell lysates or nuclear fractions. We also examined the effects of MBE and delphinidins against rotenone-induced mitochondrial dysfunction.

**Results:**

Blue light-induced cell death, increased intracellular ROS generation and mitochondrial fragmentation, decreased ATP-production coupled respiration, caused lysosomal membrane permeabilization, and increased ATF4 protein level. Treatment with MBE and its main constituents, delphinidin 3-O-sambubioside-5-O-glucoside and delphinidin 3,5-O-diglucoside, prevented these defects. Furthermore, MBE and delphinidins also protected 661W cells from rotenone-induced cell death.

**Conclusions:**

Maqui berry may be a useful protective agent for photoreceptors against the oxidative damage induced by exposure to blue light.

**Graphical abstract:**

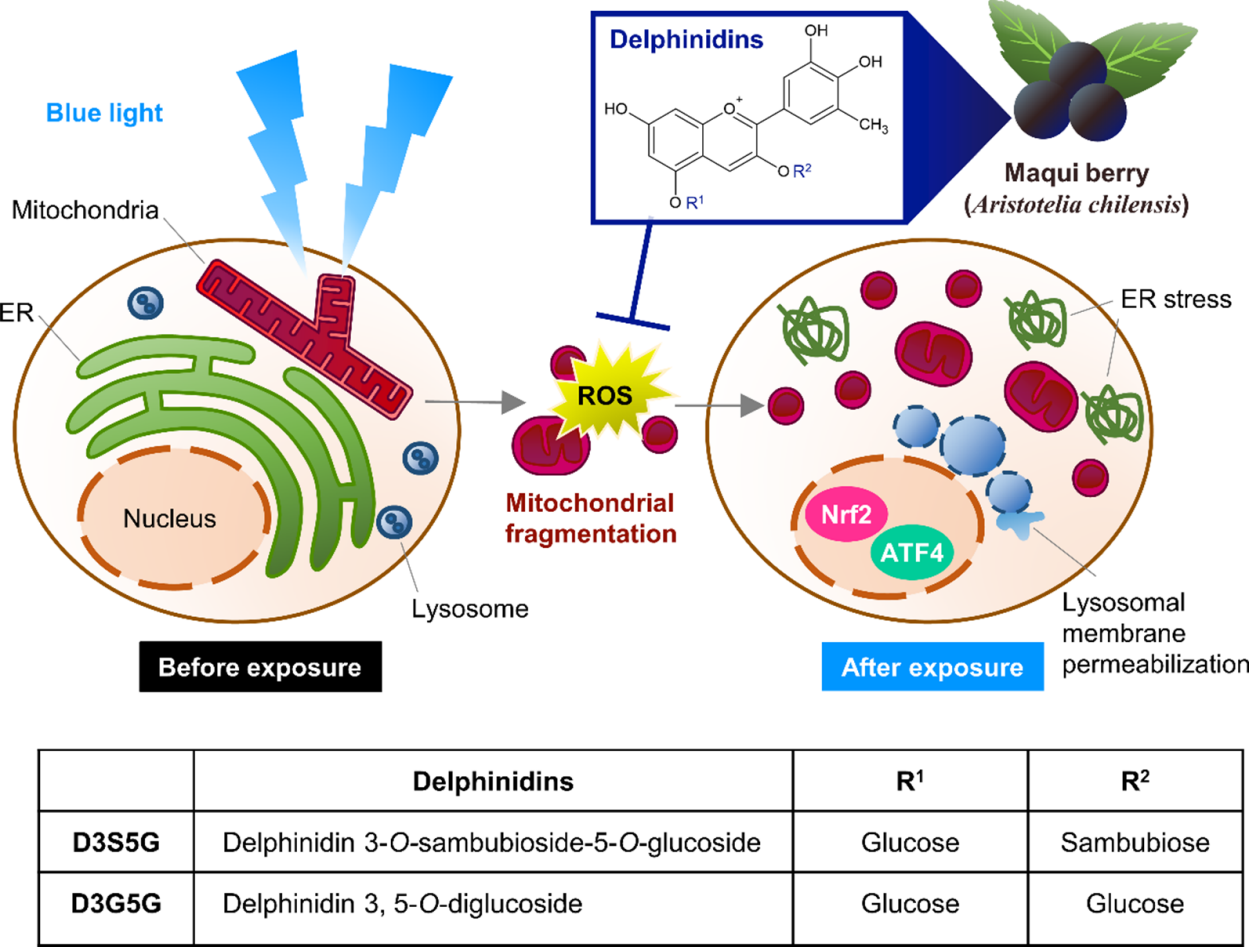

**Supplementary Information:**

The online version contains supplementary material available at 10.1186/s12906-023-04322-z.

## Introduction

Over the years, the growth of mobile technology has enabled people to access global mass communications via digital devices such as smartphones and tablets, which are equipped with light-emitting diode (LED) displays. Excessive exposure to light has been implicated in the onset and progression of retinal degenerative diseases such as age-related macular degeneration (AMD) [1]. The retina receives visible light with a wavelength of 380 to 750 nm. In particular, light of wavelengths between 380 and 500 nm is high-energy visible blue light. Blue LED light irradiation induces reactive oxygen species (ROS) production and increased endoplasmic reticulum (ER) stress, leading to apoptosis of photoreceptors [2, 3]. Although there are concerns about the impact of prolonged exposure to blue light on vision health, the mechanism of blue light-induced retinal damage has not been fully elucidated.

The retina has one of the highest metabolic demands for oxygen in the human body. Photoreceptors, the most metabolically active cells in the retina, possess an inner segment containing a large cluster of packed mitochondria. Mitochondria are a primary source of ROS as byproducts of electron transport chain activity in mitochondrial respiration [4]. Blue light causes mitochondrial DNA damage and free radical production [5], followed by excessive elevations of intracellular ROS that can trigger apoptotic cell death. Moreover, the accumulation of ROS is involved in the pathogenesis of AMD and the degeneration of retinal pigment epithelium (RPE) and photoreceptors [6]. For these reasons, antioxidants inhibiting the ROS induced by environmental stress factors such as excess light exposure have been explored, and their intake from foods has been considered beneficial for preventing retinal diseases and vision loss.

Maqui berry is a fruit of Maqui (*Aristotelia chilensis*, a plant of the Elaeocarpaceae family) and is native to central and southern Chile. It has been reported that Maqui berry extract (MBE) inhibited retinal photoreceptor damage caused by white fluorescence in a mouse-derived photoreceptor cell line [7]. Maqui berry contains high levels of delphinidins, one of the major anthocyanidins widely found in plants, which hold a 3-hydroxyl group in ring B and are known for potent antioxidant activities [8]. We also reported that anthocyanins suppress the ER stress induced by blue light irradiation [9]. However, the mechanism for the effect of MBE on blue light-induced photoreceptor damage is not fully understood.

In the present study, we aimed to investigate the effects of MBE and its constituents, delphinidins, on intracellular organelles following blue light exposure. To evaluate the effects of MBE and delphinidins on blue light-induced damage, we used an in vitro blue light exposure system with retinal cell line 661W cells and investigated cell death, the production of ROS, and changes in organelle morphology. Finally, we examined the effects of MBE and delphinidins against rotenone-induced cell death to elucidate their effects on mitochondria dysfunction.

## Materials and methods

### Reagents

MBE and delphinidins (delphinidin 3-O-sambubioside-5-O-glucoside, D3S5G; delphinidin 3,5-O-diglucoside, D3G5G) were gifted from Oryza Oil & Fat Chemical Co. Ltd. (Aichi, Japan). Primary antibodies used in this study were as follows: Lamp1 (rat, Abcam, Cambridge, MA, USA, ab25245, 1:400 for immunofluorescence staining, IF), calnexin (rabbit, Proteintech, Rosemont, IL, USA, 10427-1-AP, 1:200 for IF), activating transcription factor 4 (ATF4; rabbit, Cell Signaling Technology, Beverly, MA, USA, 11815, 1:1,000 for IB), β-actin (mouse, Sigma-Aldrich, St. Louis, MO, USA, A2228, 1:100,000 for IB), phospho-S6 ribosomal protein (p-S6RP; Ser235/Ser236, rabbit, Cell Signaling Technology, 4856, 1:1,000 for IB), S6RP (mouse, Cell Signaling Technology, 2317, 1:1,000 for IB), nuclear factor erythroid-2-related factor 2 (Nrf2; mouse, Santa Cruz, Dallas, TX, USA, sc-13032, 1:2,000 for IB), GAPDH (rabbit, Cell Signaling Technology, 2118, 1:100,000 for IB), c-Jun (rabbit, Cell Signaling Technology, 9165S, 1:100,000 for IB), translocase of outer mitochondrial membrane 20 (Tomm20; mouse, Santa Cruz, sc-17764, 1:200 for IF), and calreticulin (rabbit, Abcam, ab2907, 1:200 for IF). *N*-acetyl cysteine (NAC) was purchased from FUJIFILM-Wako Pure Chemical Corporation (Osaka, Japan). All secondary antibodies, Hoechst 33342, propidium iodide (PI), CM-H_2_DCFDA, MitoTracker Red CMXRos, and Alexa Fluor 488 annexin V were purchased from Thermo Fisher Scientific (Waltham, MA, USA). Acridine Orange (AO) was purchased from R&D systems (Minneapolis, MN, USA). Mitochondria division inhibitor 1 (Mdivi-1) and rotenone was purchased from Sigma-Aldrich (St. Louis, MO, USA).

### Cell culture and blue light irradiation

An immortalized mouse retinal cell line (661W; a kind gift from M. R. Al-Ubaidi, University of Houston, TX, USA [10]) was maintained in Dulbecco’s Modified Eagle’s Medium (DMEM; Nacalai Tesque, Kyoto, Japan) supplemented with 10% fetal bovine serum (FBS; Valeant, Costa Mesa, CA, USA) at 37 °C in a humidified atmosphere of 5% CO_2_. Blue light exposure was carried out as described previously [2]. Briefly, 661W cells were plated as follows: 3,000 cells per well in a 96-well plate; 15,000 cells per well in a 24-well plate; and 40,000 cells per well in a 6-well plate. The medium was replaced with DMEM supplemented with 1% FBS and either solvent (1 × PBS containing 0.1% dimethyl sulfoxide), MBE (1, 3, or 10 µg/mL), D3S5G (1, 3, or 10 µM), D3G5G (1, 3, or 10 µM), NAC (1 mM), or Mdivi-1 (30 µM) followed by an additional incubation at 37 °C for 1 h. The cell cultures were exposed to blue light (464 nm) from an LED source (0.38 mW/cm^2^, equivalent to 450 lx; M-Trust Co., Ltd., Hyogo, Japan) in a CO_2_ incubator under a humidified atmosphere of 5% CO_2_ at 37 °C. Then, various evaluations were performed after a certain period of time.

### Cell death assay

Cell death analyses were performed as described previously [11, 12]. In brief, Hoechst 33342 and PI (Thermo Fisher Scientific) were added to the cell cultures at the final concentration of 8.1 µM and 1.5 µM, respectively, followed by an additional incubation at 37 °C for 15 min. The images of stained nuclei were captured using a fluorescence microscope (BZ-X710; Keyence, Osaka, Japan) or a Lionheart FX Automated Microscope (BioTek Instruments, Winooski, VT, USA). The percentage of dead cells was calculated from the ratio of PI-positive cells to the number of nuclei.

### Measurement of cellular ROS production

Intracellular ROS were evaluated by a ROS indicator, CM-H_2_DCFDA (Thermo Fisher Scientific) as described previously [13]. CM-H_2_DCFDA was added to 661W cell cultures at a final concentration of 10 µM following blue light exposure for 8 h, then the fluorescence intensity (λex = 495 nm, λem = 527 nm) was measured using a microplate reader (Varioskan Flash 2.4; Thermo Fisher Scientific) immediately to obtain the signal of the background. The cells were incubated at 37 °C for an additional 1 h and subjected to the second measurement to obtain the change in fluorescence intensity. The amount of intracellular ROS was normalized by the cell numbers and shown as a relative value compared with the control group.

### Immunostaining

Immunostaining was carried out as described previously [12]. Briefly, 661W cells were cultured in a 24-well plate containing gelatin-coated coverslips (No. 1-S, a 12 mm diameter, Matsunami Glass Industry, Osaka Japan), and subjected to blue light irradiation as described previously. The cells on the coverslips were washed with PBS containing 0.2 mM CaCl_2_ and 2 mM MgCl_2_ (PBS-C/M) and fixed with 4% paraformaldehyde (PFA; Electron Microscopy Sciences, Fort Washington, PA, USA) in PBS-C/M for 10 min at room temperature. The coverslips were transferred to a clean plastic tray and then incubated with a blocking buffer (PBS-C/M containing 0.5% BSA, 0.5% saponin, 0.2 mg/mL sodium azide, and 0.2 µg/mL 4´,6-diamidino-2-phenylindole; DAPI) for 30 min, followed by incubation with primary antibodies in a blocking buffer for 1 h at room temperature. After three washes with PBS-C/M for 5 min, the samples were incubated with secondary antibodies in a blocking buffer for 30 min at room temperature. After three washes with PBS-C/M, the coverslips were mounted onto a slide with ProLong Diamond antifade reagent (Thermo Fisher Scientific). The images were acquired using Olympus FLUOVIEW FV3000 confocal laser scanning microscope (Olympus Co., Tokyo, Japan) equipped with a 60× objective lens.

### Cell lysis and immunoblotting

Cell lysis and nuclear protein extraction were performed as described previously [14]. For immunoblotting, the protein samples (2 µg per lane) were separated by electrophoresis with a 5 to 20% gradient SDS-polyacrylamide gel (SuperSep Ace, FUJIFILM-Wako) and transferred to Polyvinylidene difluoride membranes (Immobilon P; Millipore, Billerica, MA, USA). The membranes were incubated with Blocking One-P (Nacalai Tesque) for 1 h, followed by incubation with the primary antibody in Can get signal solution 1 (Toyobo) overnight at 4 °C. After washing three times with TBS containing 0.05% Tween-20, the membrane was incubated with the secondary antibody in Can get signal solution 2 (Toyobo) for 2 h at room temperature with gentle agitation. Finally, the signals were visualized by ImmunoStar LD (FUJIFILM-Wako) and detected using Amersham Imager 680 (GE Healthcare, Chicago, IL, USA). The expression level of each protein was determined by quantifying the intensity of the bands using Amersham Imager 680 Analysis software (GE Healthcare).

#### Oxygen consumption rate (OCR) measurement

Cell Mito Stress Test (Agilent Technologies, Santa Clara, CA, USA) was performed according to the manufacturer’s instructions. Briefly, 661W cells were plated at 10,000 cells per well into Seahorse XF HS 8-well plates (Agilent Technologies) 24 h prior to the assay. The medium was replaced with fresh DMEM containing 1% FBS and incubated for another 1 h, followed by the exposure to blue light for 8 h. Immediately after irradiation, the medium was replaced by Seahorse XF DMEM Medium supplemented with 10 mM glucose, 1 mM pyruvate, and 2 mM L-glutamine (Agilent Technologies), and incubated for 1 h at 37 °C without CO_2_ supplement. Finally, the plates were transferred to a Seahorse XF HS Mini Analyzer (Agilent Technologies), and a Cell Mito Stress Test was performed (Agilent Technologies; 1.5 µM oligomycin A, 1.0 µM FCCP, and a mixture of 0.5 µM rotenone and 0.5 µM antimycin). After the OCR measurement, the cell death assay was performed using a Lionheart FX Automated Microscope (BioTek Instruments) as described previously. Data analyses were conducted using Agilent Seahorse Analytics (Agilent Technologies). Each OCR value was normalized by the number of PI-negative viable cells.

### Mitochondrial morphological analysis

MitoTracker staining and morphological analysis were done as reported previously with minor modifications [15]. Briefly, 661W cells cultured in a 6-well plate (Corning) containing coverslips (No. 1-S, Matsunami Glass Industry, Osaka, Japan) coated with 0.2% porcine skin gelatin (Sigma-Aldrich, G2500) were incubated with 100 nM MitoTracker Red CMXRos (Thermo Fisher Scientific) at 37 °C for 15 min and then fixed with 4% PFA (Electron Microscopy Sciences) in PBS-C/M for 10 min at room temperature. The fixed cells were stained with 0.2 µg/mL DAPI (Biotium, Hayward, CA, USA) in PBS/C-M for 30 min at room temperature. The stained samples were mounted with ProLong Diamond Antifade Mountant (Thermo Fisher Scientific) and subjected to confocal microscopy using an FV3000 microscope (Olympus Co.) equipped with a 60× objective oil lens. Mitochondrial morphological analysis was carried out on ImageJ Ver. 1.53c (National Institutes of Health, Bethesda, MD, USA). The numbers of mitochondria branches were obtained from skeletonized images using Skeleton Plugin. Thirty-six cells from three independent experiments were analyzed and displayed as scatter plots.

To analyze the mitochondrial fragmentation and apoptosis simultaneously, cells were stained with MitoTracker Red CMXRos (Thermo Fisher Scientific) and Alexa Fluor 488 annexin V (Thermo Fisher Scientific), according to the manufacturer’s instructions. Briefly, 661W cells on a gelatin-coated coverslip were rinsed with pre-warmed Annexin-binding buffer (10 mM HEPES, 140 mM NaCl, 2.5 mM CaCl_2_, pH 7.4) once, and incubated with 100 nM MitoTracker Red and Alexa Fluor 488 annexin V at 37 °C for 15 min. The stained cells were subjected to fixation and DAPI staining as described previously. Images were obtained using an FV3000 confocal microscope (Olympus Co.) with a 60× objective oil lens.

### AO staining

In order to visualize acidic compartments, live cell imaging was performed for 661W cells stained with AO (R&D systems). 661W cells were plated in a multi-well glass bottom dish (Matsunami Glass Industry) at a density of 10,000 cells per well and incubated overnight at 37 °C. The medium was replaced with DMEM containing 1% FBS, and the cells were incubated with either 10 µg/mL MBE, 10 µM D3S5G or 10 µM D3G5G for 1 h at 37 °C, followed by exposure to blue light for 8 h. AO was added to a final concentration of 1 mg/mL, and the cells were incubated for 20 min at 37 °C. Finally, the medium was changed to prewarmed Live Cell Imaging Solution (LCIS, Thermo Fisher Scientific) containing 5.5 mM glucose and 1% FBS, and cells were subjected to live cell imaging using a 40× oil lens on an FV3000 microscope (Olympus Co.) equipped with a stage top incubator (Tokai Hit Co., Shizuoka, Japan). AO green and red fluorescence intensities were measured using ImageJ Ver. 1.53c (National Institutes of Health) for 24 cells each in four independent experiments to obtain the intensity ratio of green vs. red.

### Statistics

Data are presented as the mean ± standard error of the mean (SEM). Statistical analyses were performed using a two-tailed Student’s *t*-test, Tukey’s test, or Dunnett’s test using the Statistical Package for the Social Sciences 15.0 J for Windows software (SPSS Japan Inc., Tokyo, Japan). *P*-values < 0.05 were considered statistically significant.

## Results

### Effect of MBE and its constituents on blue light-induced photoreceptor damage

**Fig. 1:**
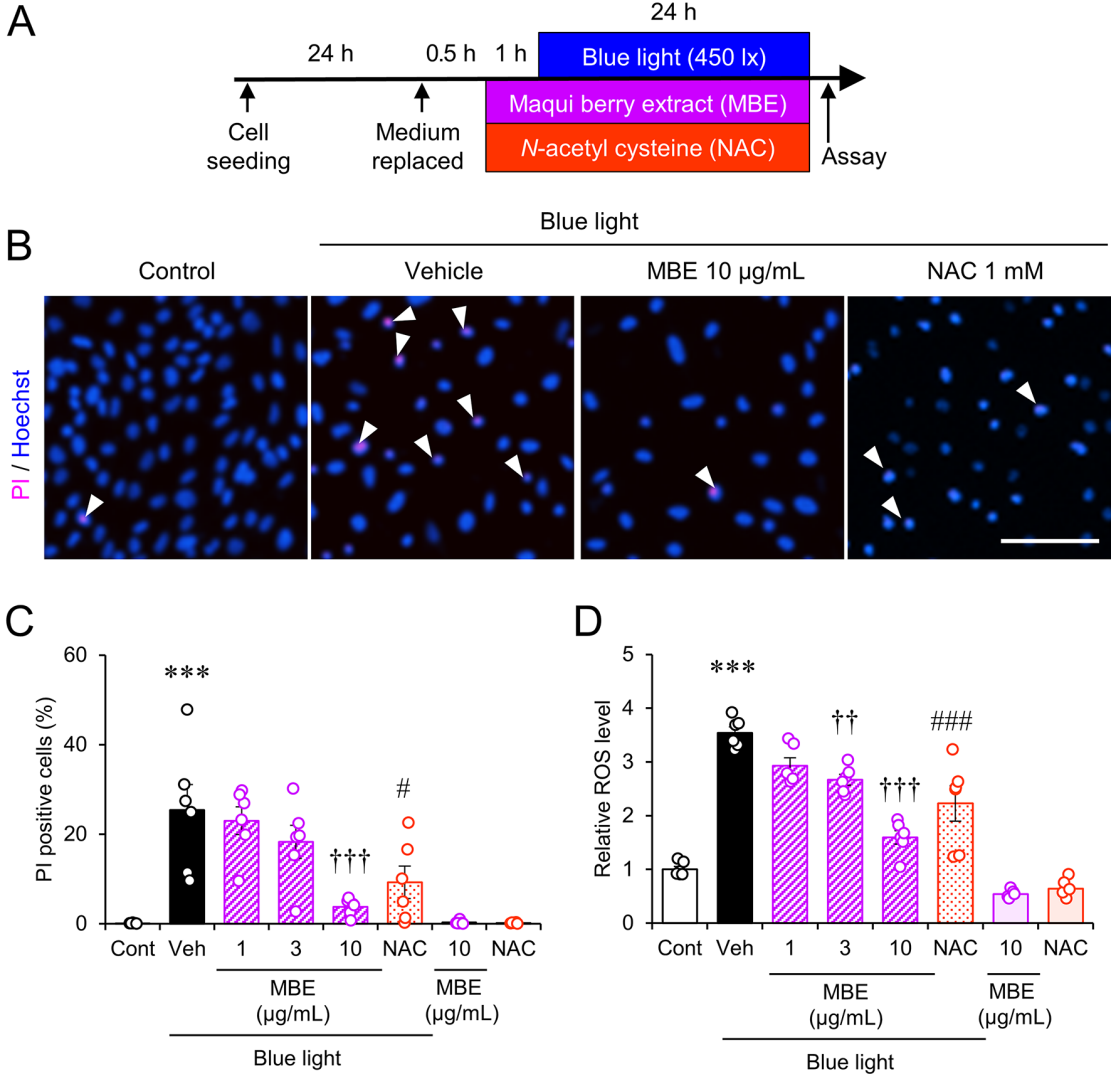
MBE and NAC suppressed the elevation of cell death and ROS production in 661W cells exposed to blue light. (**A**) The diagram depicts the timeline of MBE treatment and blue light exposure. Cells were incubated in the presence of MBE (1, 3, 10 µg/mL) or *N-*acetyl cysteine (NAC, 1 mM) with or without blue light irradiation (450 lx) for 24 h, followed by cell death assay and ROS measurement. (**B**) Representative images of Hoechst 33342 (blue) and PI (magenta) staining. Arrowheads indicate PI-positive nuclei. Bar = 100 μm. (**C**) The cell death ratio is the percentage of PI-positive to Hoechst 33342-positive cells. (**D**) Relative ROS production determined by fluorescence intensity of CM-H_2_DCFDA is presented as relative units compared to the control. Bars are shown as the mean ± S.E.M (n = 6). ***P < 0.001, Tukey’s test vs. control (Cont); ^††^*P* < 0.01, ^†††^*P* < 0.001, Dunnett’s test vs. vehicle (Veh); ^#^*P* < 0.05, ^###^*P* < 0.001, Dunnett’s vs. Veh

To investigate the effect of MBE on the photodamage induced by blue light, mouse retina-derived 661W cells were pre-treated with MBE at 1, 3, or 10 µg/mL for 1 h and exposed to 450 lx blue light for an additional 24 h (Fig. 1A). Double staining with Hoechst 33342 and PI showed PI-positive dead cells (Fig. 1B). The ratio of PI-positive cells increased to 25.4 ± 6.3% when 661W cells were exposed to blue light for 24 h (Fig. 1B, C). Blue light-induced cell death was significantly reduced to 3.7 ± 0.8% when cells were incubated with MBE at 10 µg/mL (Fig. 1B, C). Blue light irradiation is known to stimulate the production of ROS [2]. The intracellular ROS level increased to 3.54 ± 0.11-fold compared to the control group. In contrast, the increase of ROS was suppressed in the presence of 3 and 10 µg/mL of MBE (Fig. 1D). The treatment of 1 mM of *N*-acetyl cysteine (NAC) also suppressed blue-light-induced cell death and ROS production (Fig. 1B, C, D). Taken together, the compounds possessing antioxidant properties like MBE and NAC protected 661W cells from blue-light-induced cell death, and it may be through the suppression of the increase of ROS production.

**Fig. 2:**
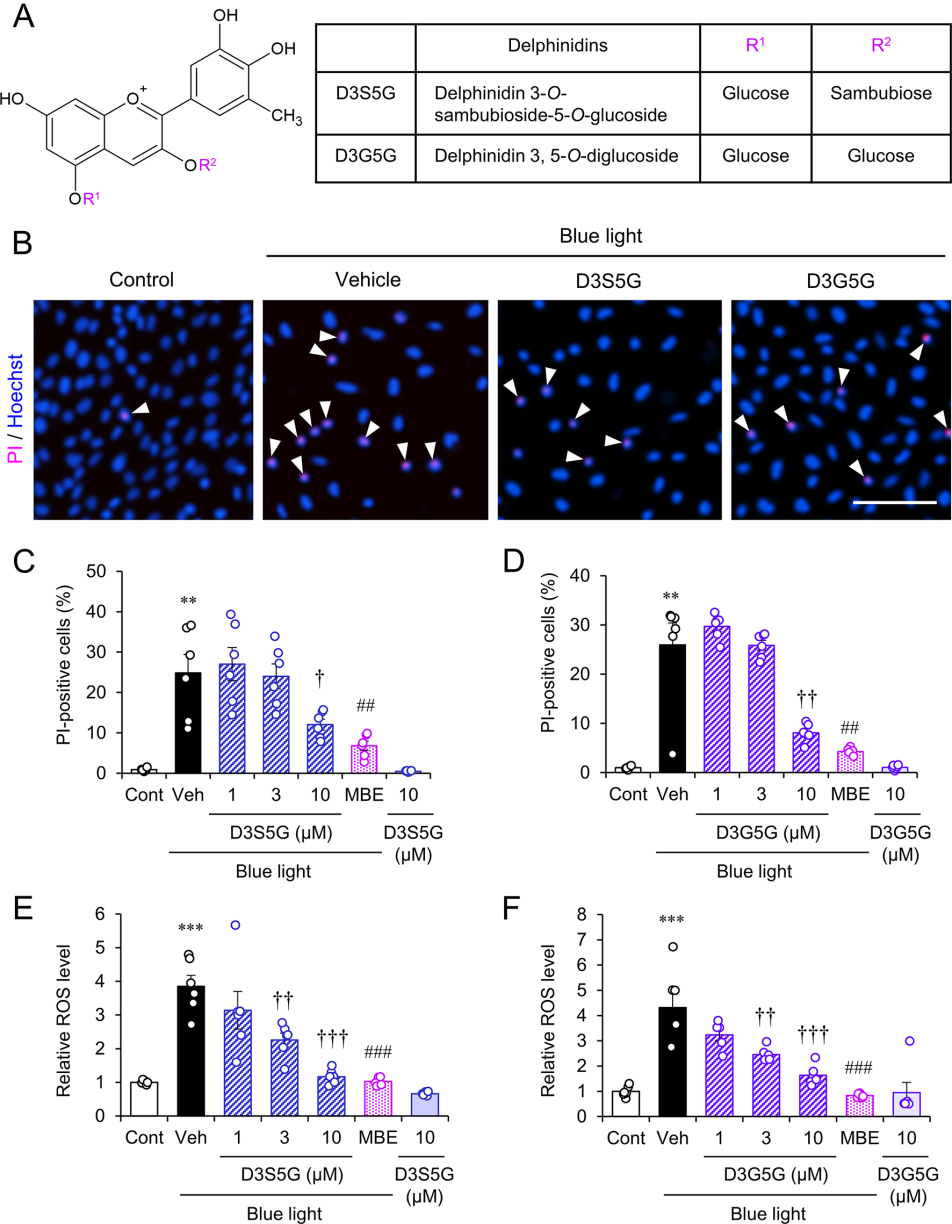
Delphinidins inhibited the blue light-induced cell damage in 661W cells. (**A**) Chemical structures of D3S5G and D3G5G. (**B**) Representative images of Hoechst 33342 (blue) and PI (magenta) staining in 661W cells incubated with or without 10 µM D3S5G or 10 µM D3G5G following blue light exposure. Arrowheads indicate PI-positive nuclei. Bar = 100 μm. (**C**, **D**) Quantification of the cell death ratio in 661W cells with D3S5G (**C**) or D3G5G (**D**). (**E**, **F**) Relative levels of ROS in 661W cells with D3S5G (**E**) or D3G5G (**F**). MBE was added at 10 µg/mL as a control. Data are shown as the mean ± S.E.M (n = 6) **P < 0.01, ***P < 0.001, Tukey’s test vs. control (Cont); ^†^*P* < 0.05, ^††^*P* < 0.01, ^†††^*P* < 0.001, Dunnett’s test vs. vehicle (Veh); ^##^*P* < 0.01, ^###^*P* < 0.001, Dunnett’s vs. Veh

Previously, we identified eight anthocyanin components of MBE using HPLC analysis [7] and found that delphinidins are enriched in MBE. Two types of delphinidins, delphinidin 3-O-sambubioside-5-O-glucoside (D3S5G) and delphinidin 3,5-O-diglucoside (D3G5G), are contained in MBE at 7.1 and 14.3%, respectively [7]. Delphinidins are known for their high antioxidant capacity due to the large number of hydroxyl groups in their structure (Fig. 2A). The increased cell death ratio following blue light exposure was sustained in the groups treated with either 10 µM D3S5G (Fig. 2B, C) or 10 µM D3G5G (Fig. 2B, D) to a similar extent as with MBE. Moreover, the elevated ROS level following blue light exposure was mitigated in the presence of D3S5G or D3G5G at concentrations of 3 and 10 µM (Fig. 2E, F). These results indicate that D3S5G and D3G5G have independent protective effects against blue light-induced oxidative damage in 661W cells.

### Expression of stress response factors under blue light irradiation

**Fig. 3:**
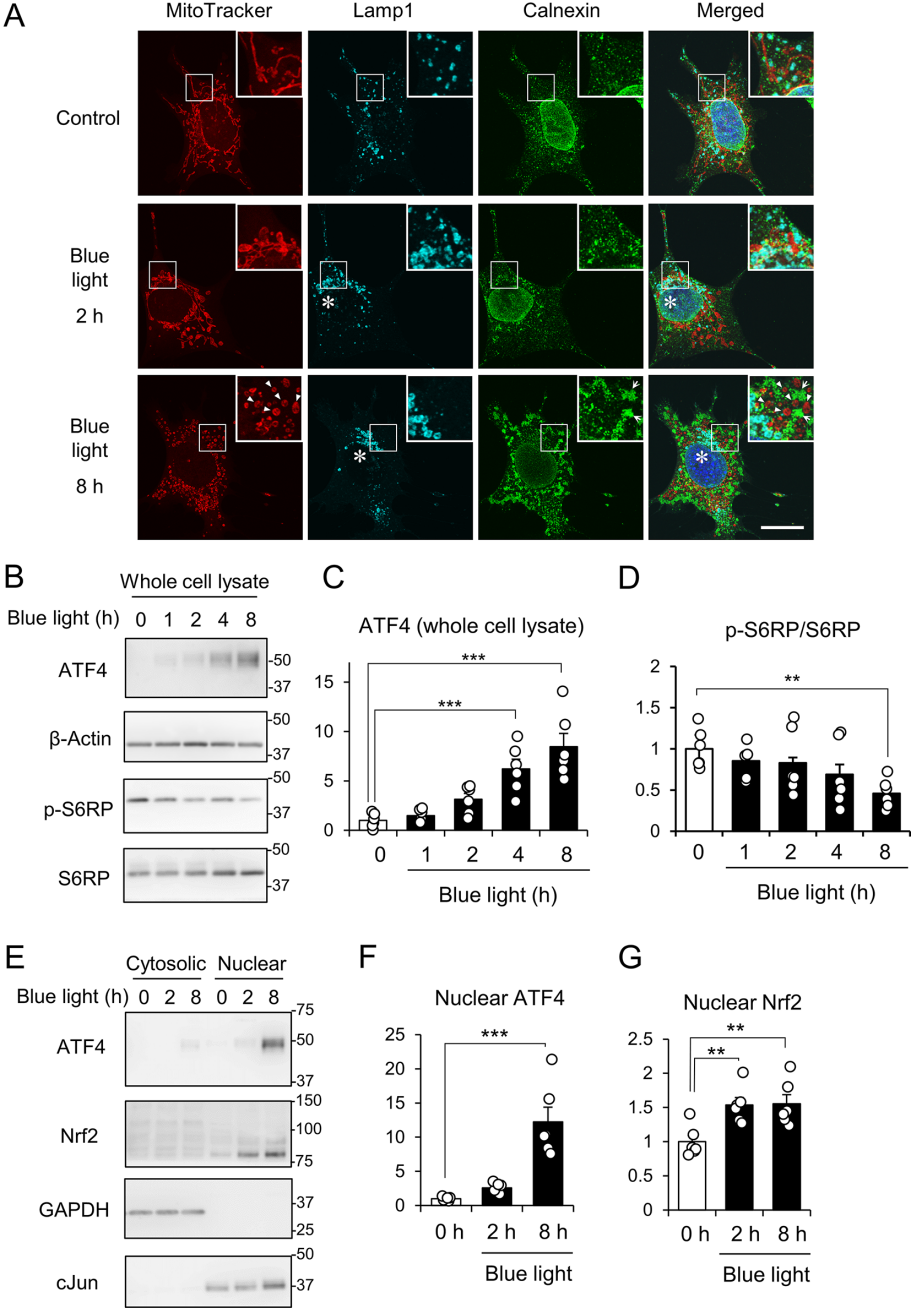
Blue light-induced morphological changes of intracellular organelles and the subsequent activation of stress signaling pathways in 661W cells. (**A**) Representative images of mitochondria (MitoTracker, red), lysosomes (Lamp1, cyan), ER (calnexin, green), and nuclei (DAPI, blue) in 661W cells incubated without (Control) or with either 2 or 8 h of exposure to blue light. Enlarged views of the boxed regions are shown in the upper right corners. Arrowheads indicate fragmented mitochondria. Bar = 20 μm. (**B**) Representative images of immunoblots in whole cell lysates obtained from 661W cells at the indicated times under the condition with blue light irradiation. (**C**, **D**) The graph represents the relative protein level of ATF4 (**C**, normalized by β-actin) and the phosphorylation of S6 ribosomal protein (S6RP; **D**) (**E**) Representative images of immunoblots in the cytosolic and nuclear fractions. (**F**, **G**) The graph displays the relative levels of ATF4 (**F**) and Nrf2 (**G**), which are normalized by cJun. Bar graph shows the mean ± SEM. (n = 6). **P < 0.01, ***P < 0.001, Tukey’s test vs. 0 h

Intracellular organelles such as mitochondria and ER are known to be associated with photoreceptor damage caused by blue light [2, 3]. Recently, it has been shown that lysosomal distribution and morphology are also altered following blue light exposure [12]. As was reported previously, aberrantly enlarged lysosomes and aggregation of the ER were observed at 8 h of blue light irradiation (Fig. 3A). Moreover, mitochondria became a round shape in 661W cells irradiated with blue light for 2 or 8 h, instead of tubular structures found in the control group (Fig. 3A). The changes in mitochondria were found earlier than for other organelles, suggesting that mitochondria are more susceptible to blue light damage. Next, we explored the signaling pathway linked to each organelle. ATF4 is a transcription factor whose expression is enhanced by ER stress. As reported previously [3], blue light exposure for 4 and 8 h increased the protein level of ATF4 (Fig. 3B, C). Lysosomes are required for the mechanistic target of rapamycin complex 1 (mTORC1) signaling [16]. The phosphorylation of S6RP, downstream of the mTORC1 signaling pathway, was suppressed after 8 h of irradiation with blue light (Fig. 3B, D). The nuclear fraction of ATF4 was increased at 8 h but not at 2 h following blue light exposure (Fig. 3E, F). On the other hand, the Nrf2 level at the nuclear fraction was elevated even at 2 h blue light irradiation (Fig. 3E, G), indicating that the oxidative stress response associated with Nrf2 was stimulated earlier than for the other signaling pathways.

### MBE and anthocyanins can improve blue light-induced defects in mitochondrial function and morphology of 661W cells

**Fig. 4:**
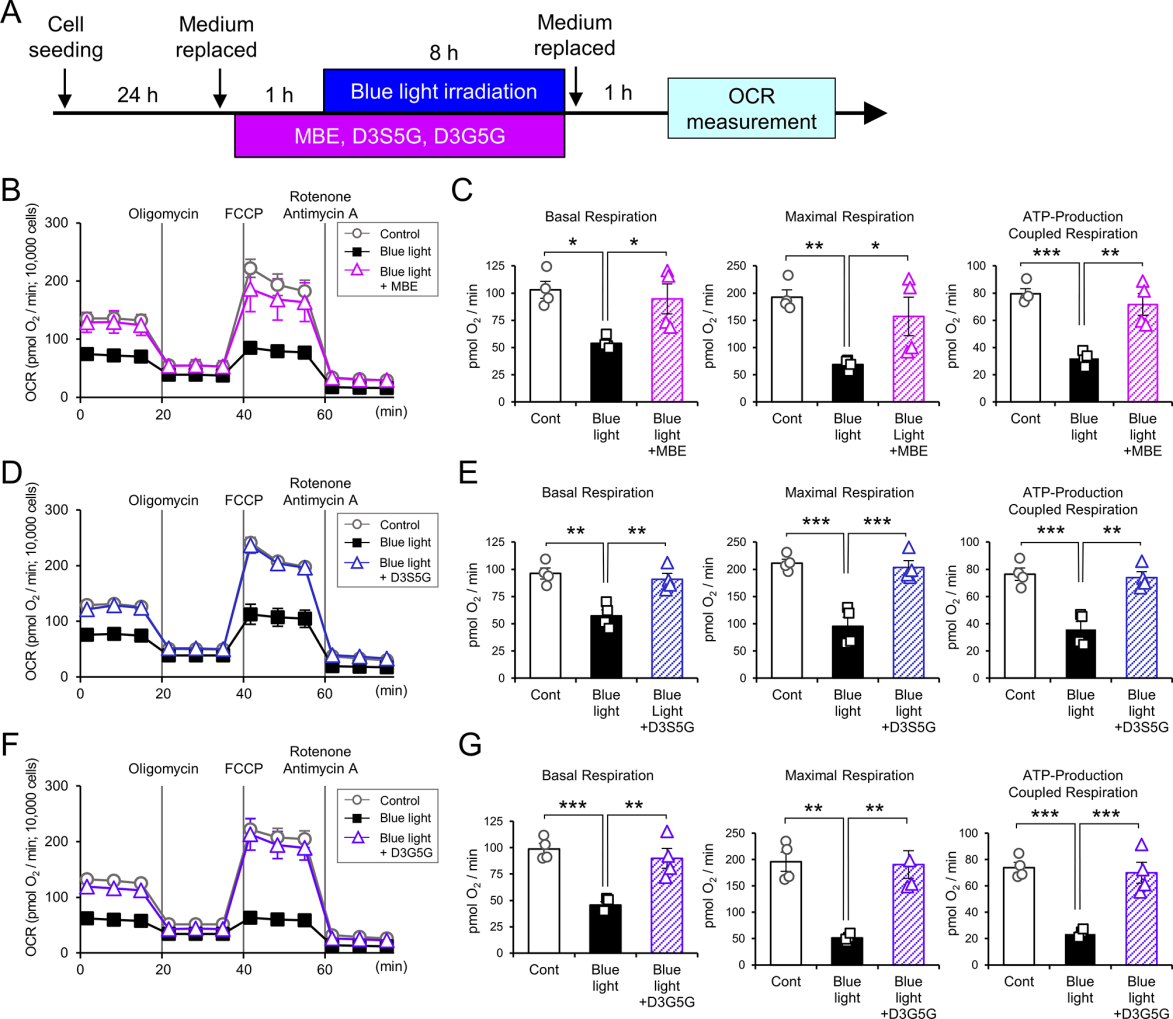
MBE and delphinidins ameliorate the decrease in oxygen consumption rates of 661W exposed blue light. (**A**) A schematic diagram of the experimental design for the Mito Stress assay used in this study. (**B**, **D**, **F**) The representative traces of OCR in 661W cells over time (min) following blue light irradiation without or with 10 µg/mL MBE (**B**), 10 µM D3S5G (**D**), or 10 µM D3G5G (**F**). (**C**, **E**, **G**) The graph shows basal respiration, maximal respiration, and ATP-production coupled respiration. Bar graph represents the mean ± SEM (n = 4). ^*^P < 0.05, ^**^P < 0.01, ^***^P < 0.001 Tukey’s test vs. Blue light

Mitochondria are responsible for cellular energy synthesis, achieved via oxidative phosphorylation with the electron transport chain and ATP synthase at the inner membrane. We examined whether blue light affects the function of mitochondria. The Mito Stress Test was carried out following the exposure of cells to blue light for 8 h (Fig. 4A). The levels of OCR were reduced by blue light exposure (Fig. 4B, D, F). The basal respiration, maximal respiration, and ATP-production coupled respiration decreased in 661W cells exposed to blue light (Fig. 4C, E, G). Next, we examined the effects of MBE and delphinidins on OCR in 661W cells exposed to blue light. The levels of OCR were recovered in the presence of 10 µg/mL MBE, 10 µM D3S5G, or 10 µM D3G5G (Fig. 4B, D, F). The basal respiration, maximal respiration, and ATP-production coupled respiration were significantly improved when 661W cells were treated with 10 µg/mL MBE, 10 µM D3S5G, or 10 µM D3G5G (Fig. 4C, E, G). Taken together, MBE and delphinidin treatment can prevent the deterioration of mitochondrial activity under the condition of blue light-induced oxidative stress.

**Fig. 5:**
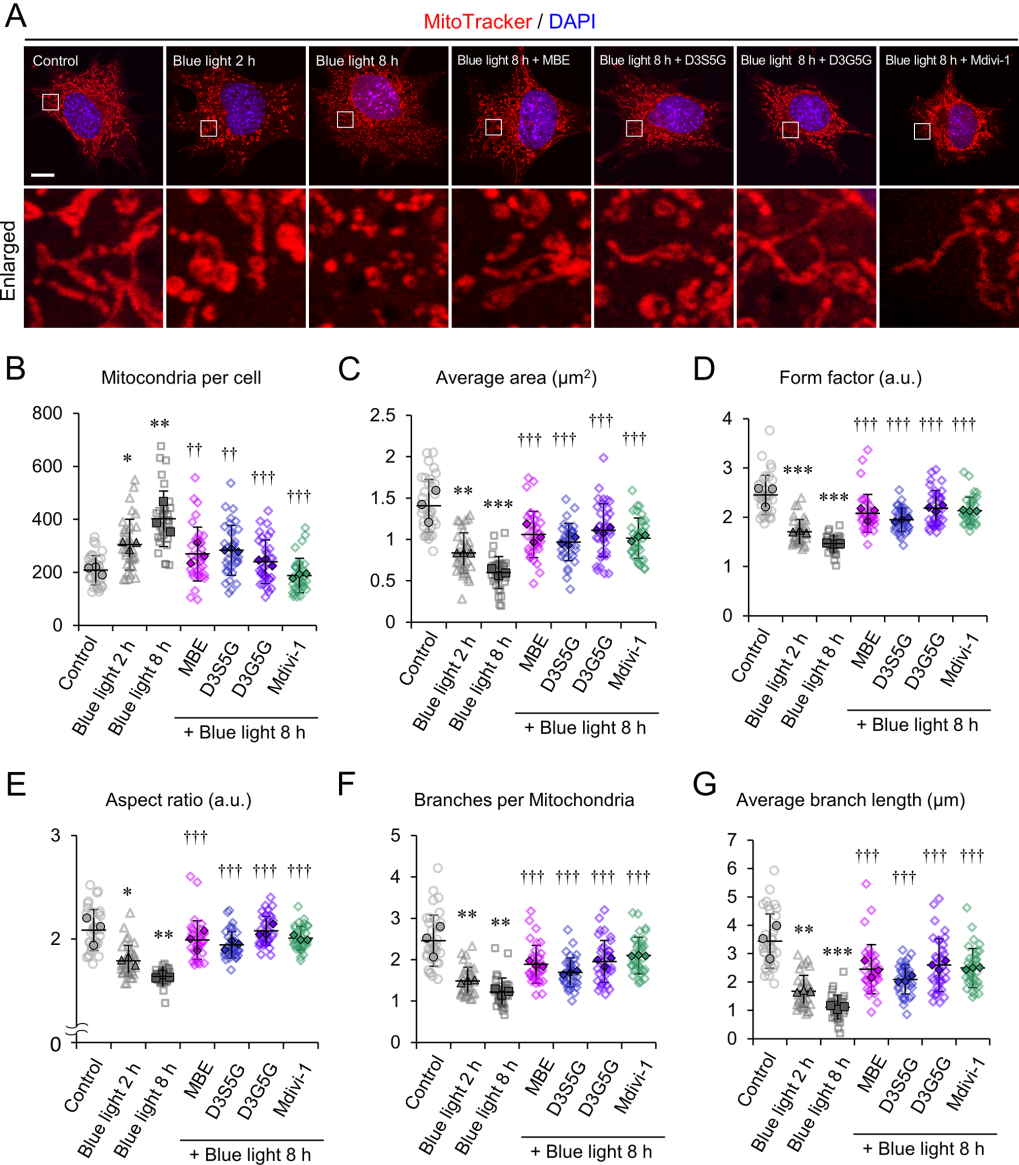
MBE, delphinidins, and Mdivi-1 prevented mitochondrial fragmentation induced by blue light exposure. (**A**) Representative images of 661W cells stained with MitoTracker (red) and DAPI (blue) following blue light exposure for the indicated times with or without 10 µg/mL MBE, 10 µM D3S5G, 10 µM D3G5G, or 30 µM mitochondrial division inhibitor 1 (Mdivi-1). Enlarged views of the indicated area are shown at the bottom. Bar = 20 μm. (**B**–**G**) Quantitative results for the number of mitochondria per cell (**B**) and average area (**C**), form factor (**D**), aspect ratio (**E**), branch number (**F**), and branch length (**G**) of each mitochondrion. A total of 36 cells from three independent experiments were analyzed. The average of each experiment is shown by a solid line. **P < 0.05, **P < 0.01, ***P < 0.001, Tukey’s test vs. control (Cont); ^††^*P* < 0.01, ^†††^*P* < 0.001, Dunnett’s test vs. vehicle (Veh)

The mitochondrial network is highly dynamic, which is linked to mitochondrial function and ROS production [17]. To investigate the effects of MBE and delphinidins on mitochondrial dynamics, the shape of the mitochondrial network was analyzed by staining with MitoTracker. As shown in Fig. 3A, mitochondrial fragmentation was observed at 2 and 8 h of exposure to blue light (Fig. 5A). Morphological analysis revealed that blue light irradiation increased the number of mitochondria per cell and decreased the mean surface area (Fig. 5B, C). Furthermore, blue light irradiation decreased the form factor and the aspect ratio of each mitochondrion (Fig. 5D, E). Consistently, the number and the average length of branches were reduced by blue light irradiation (Fig. 5F, G). When cells were treated with 10 µg/mL MBE, 10 µM D3G5G, or 10 µM D3S5G, these morphological changes were not so prominent as the vehicle group with 8 h of blue light irradiation (Fig. 5B). Each value of the quantitative analyses was partially improved in the presence of MBE or delphinidins (Fig. 5C–G). We also investigated the effect of mitochondrial division inhibitor 1 (Mdivi-1) on the mitochondrial morphology following after blue light exposure. When 661W cells were treated with 30 µM Mdivi-1, the structure of mitochondria was preserved to some extent after the exposure to blue light for 8 h (Fig. 5A, C-G). Notably, the number of mitochondria was maintained at a similar level to the control group even after blue light exposure (Fig. 5B).

**Fig. 6:**
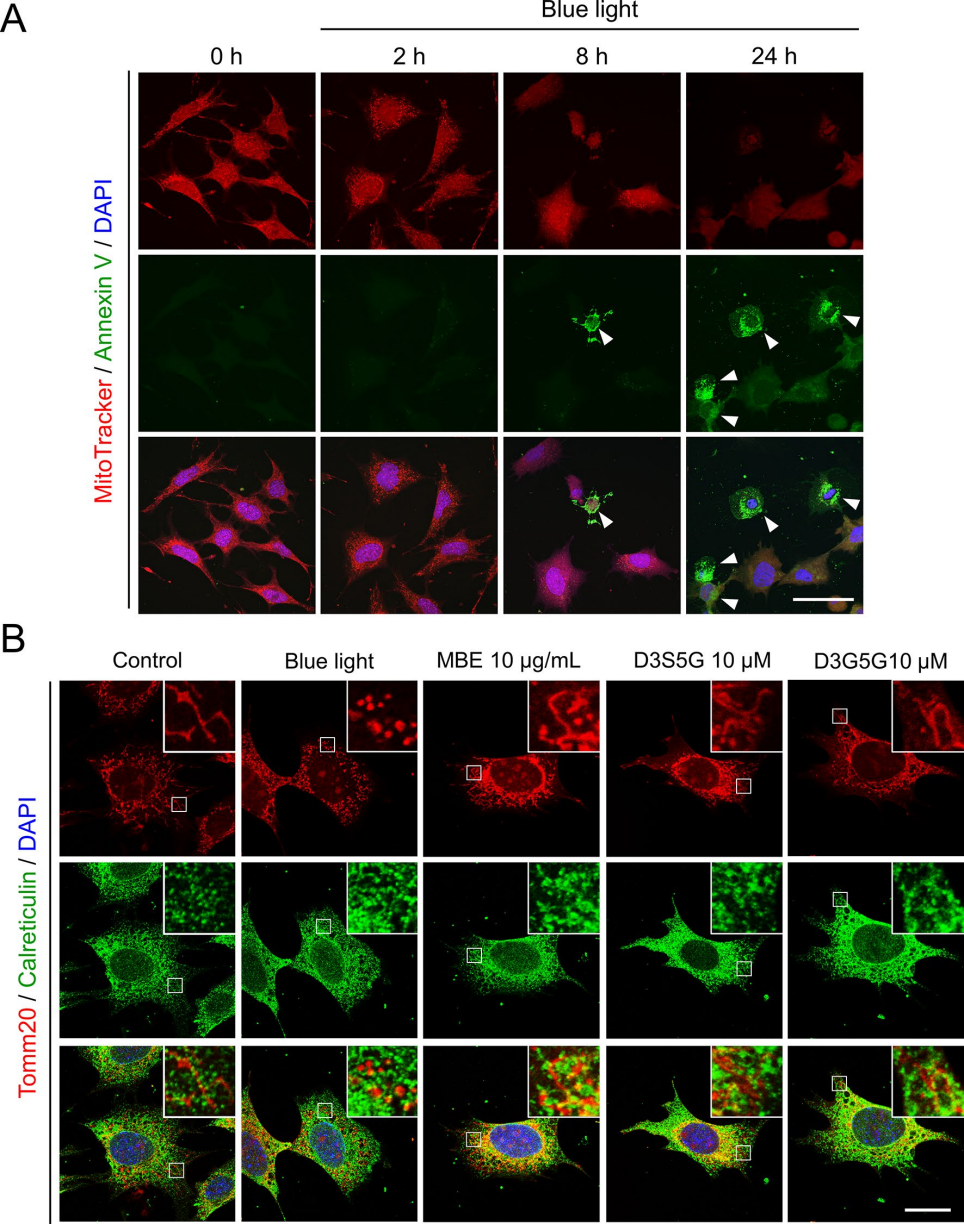
Blue light-induced morphological changes of mitochondria preceded apoptosis. (**A**) Representative images of mitochondria (MitoTracker, red), externalized phosphatidylserine (annexin V, green), and nuclei (DAPI, blue) in 661W cells exposed to blue light for the indicated time. Arrowheads indicate apoptotic cells. (**B**) Representative images of mitochondria (Tomm20, red), ER (calreticulin, green), and nuclei (DAPI, blue) in 661W cells exposed to blue light for 8 h with 10 µg/mL MBE, 10 µM D3S5G, or 10 µM D3G5G. Enlarged views of the boxed regions are shown in the upper right corners. Bars = 50 μm (A), 20 μm (**B**)

Next, we evaluated the mitochondrial morphology together with apoptosis by double staining with MitoTracker and annexin V. Mitochondrial fragmentation were observed at the time of 2 h after blue light exposure without the detection of annexin-V-positive cells (Fig. 6A). A small number of apoptotic cells were seen after 8 h of blue light exposure (Fig. 6A), in which cells only the small puncta of mitochondria remained. The immunostaining for the mitochondrial marker Tomm20 enabled to visualize the structure of mitochondria and also showed the mitochondrial fragmentation following exposure to blue light (Fig. 6B). The treatment of MBE and delphinidins preserved mitochondrial structures and their interaction with the ER in 661W cells following after blue light exposure (Fig. 6B). In conclusion, treatment with MBE and delphinidins can protect mitochondrial structures and function from blue light.

### MBE and anthocyanins ameliorate the damage to intracellular organelles and stress responses induced by blue light irradiation

**Fig. 7:**
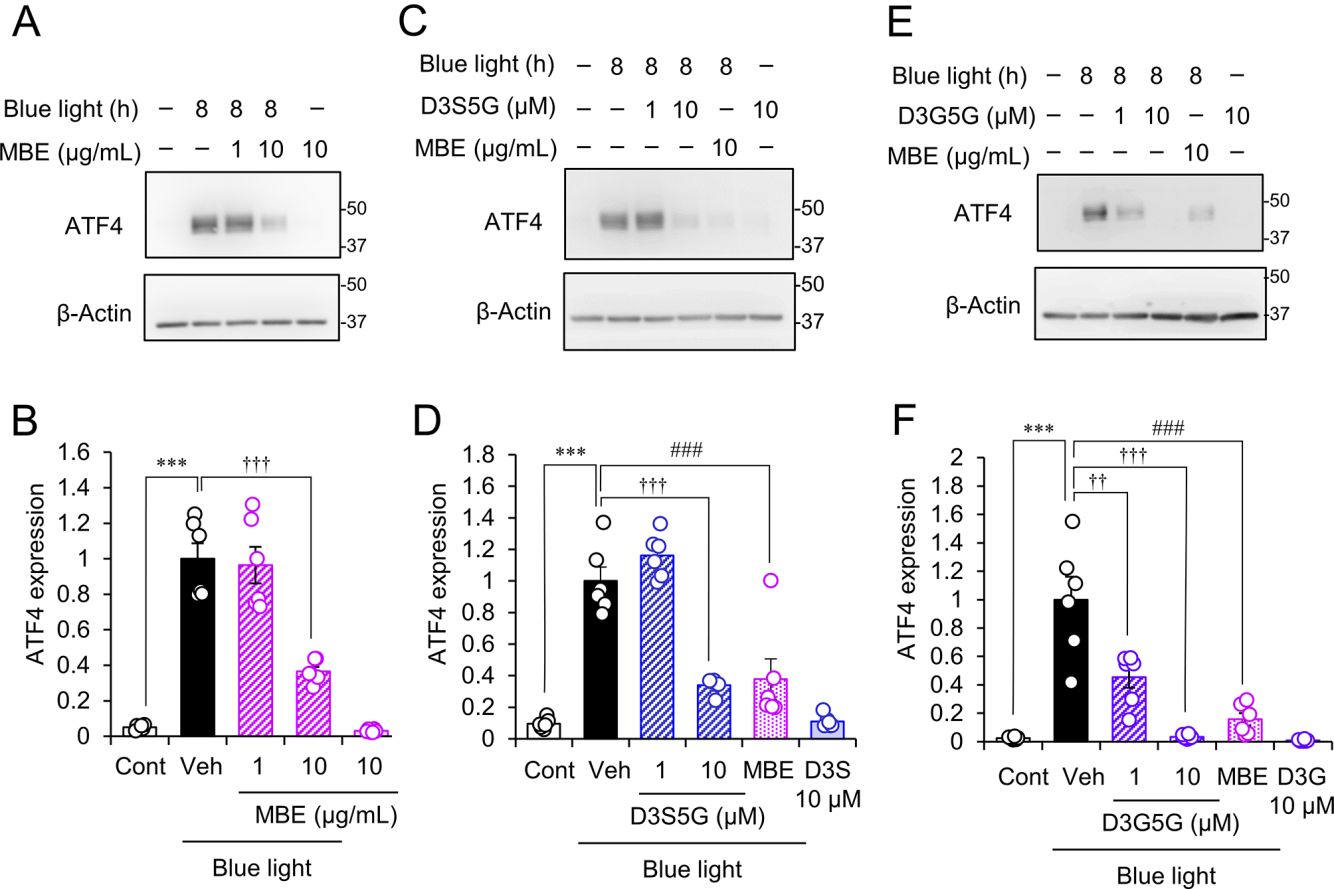
MBE and delphinidins suppressed the increase of ATF4 expression in 661W cells exposed to blue light. (**A**, **C**, **E**) Representative images of immunoblots of whole cell lysates obtained from 661W cells incubated with MBE (**A**, **C**, **E**), D3S5G (**C**), and D3G5G (**E**) for the indicated times exposed to blue light. (**B**, **D**, **F**) The graph represents the relative protein level of ATF4 normalized to that of β-actin (Vehicle = 1). Bar graphs show the mean ± SEM. (n = 6). ***P < 0.001, Tukey’s test vs. control (Cont); ^††^*P* < 0.01, ^†††^*P* < 0.001, Dunnett’s test vs. vehicle (Veh); ^###^*P* < 0.001, Dunnett’s vs. Veh

Subsequently, we assessed the effect of MBE and delphinidins on the blue light-induced increase of ATF4. Immunoblotting of whole cell lysates showed that 10 µg/mL of MBE attenuated the increase of ATF4 protein levels at 8 h of exposure to blue light (Fig. 7A, B). Quantification revealed that ATF4 was significantly suppressed to 36.6 ± 2.6% in the 10 µg/mL MBE treatment group compared to the vehicle group (Fig. 7B). ATF4 expression was decreased in the cells treated with the delphinidin D3S5G at 10 µM to a similar extent as the group of 10 µg/mL MBE (Fig. 7C, D). Of note, D3G5G suppressed the ATF4 expression even at 1 µM (Fig. 7E, F). Altogether, 10 µg/mL of MBE and 10 µM of delphinidins can inhibit the ER stress induced by blue light.

**Fig. 8:**
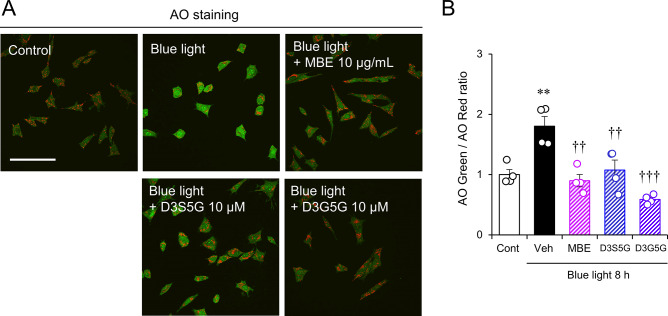
MBE and delphinidins protect lysosomes against blue light-induced damage. (**A**) Representative images of AO staining in 661W cells exposed to 8 h of blue light with MBE, D3S5G, and D3G5G. The fluorescent signals of AO in lysosomes and in the cytosol and nucleus are shown in red and green, respectively. Bar = 100 μm. (**B**) This graph shows the rate of green and red fluorescence emission. Data are expressed as the mean ± S.E.M. (n = 4). **P < 0.01, Student’s *t*-test. vs. control (Cont); ^††^*P* < 0.01, ^†††^*P* < 0.001, Dunnett’s test vs. vehicle (Veh)

It has been reported that blue light irradiation causes lysosomal membrane permeability (LMP) [12]. We explored LMP using the ratio of red to green fluorescence luminance of AO as an indicator of membrane permeability. As reported previously, LMP occurred following 8 h of exposure to blue light (Fig. 8A, B). The LMP was reduced by the addition of MBE, D3S5G, and D3G5G (Fig. 8A, B), suggesting that MBE and delphinidins can protect lysosomal membrane integrity from the oxidative stress induced by blue light.

### Protective action of MBE and its components against rotenone-induced mitochondrial damage

**Fig. 9:**
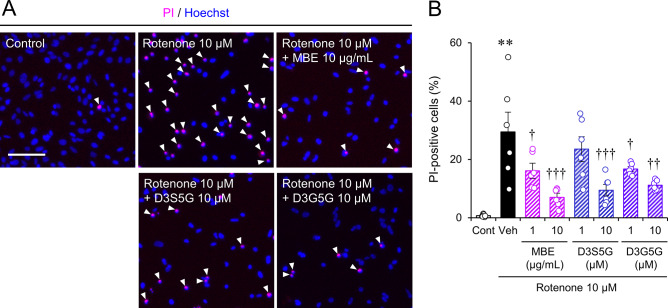
MBE and delphinidins ameliorated cell death induced by inhibiting complex I of the mitochondrial respiratory chain in 661W cells. (**A**) Representative images of Hoechst 33342 (blue) and PI (magenta) staining in 661W cells incubated with 10 µM rotenone and/or with MBE, D3S5G, and D3G5G for 24 h. Arrowheads indicate PI-positive nuclei. Bar = 100 μm. (**B**) Quantification of the cell death ratio. Data are shown as the mean ± S.E.M (n = 6). **P < 0.01, Student’s *t*-test. vs. control (Cont); ^†^*P* < 0.05, ^††^*P* < 0.01, ^†††^*P* < 0.001, Dunnett’s test vs. vehicle (Veh)

Rotenone is known to inhibit respiratory chain complex I and cause dysfunction in mitochondria [18]. We investigated the protective effects of MBE and delphinidins against rotenone-induced mitochondrial dysfunction. In the rotenone-treated group, PI-positive dead cells increased to 29.5 ± 6.8% (Fig. 9A, B), whereas the dead cell ratio was 7.0 ± 1.4%, 9.5 ± 2.0%, and 11.2 ± 0.8% in the groups treated with 10 µg/mL MBE, 10 µM D3S5G, and 10 µM D3G5G, respectively (Fig. 9B). These results indicate that MBE and the delphinidins can directly act on mitochondria and protect photoreceptor cells from the cell death induced by mitochondrial dysfunction.

## Discussion

In this study, we explored the changes of intracellular organelles in 661W cells exposed to blue light and found that blue light induced mitochondrial fragmentation and a decrease of ATP-production coupled respiration (Figs. 3, 4, 5 and 6). Treatment with MBE and its major constituents, delphinidins D3S5G and D3G5G, were able to prevent this damage to mitochondrial structures and function (Figs. 4, 5 and 6), lysosomal membrane permeability (Fig. 8), and cell death (Figs. 1 and 2) in 661W cells exposed to blue light. MBE and delphinidins suppressed the ATF4 expression induced by blue light irradiation (Fig. 7). Finally, MBE and delphinidins also protected 661W cells from rotenone-induced cell death, suggesting that they might act on mitochondria directly and prevent the subcellular damages induced by mitochondrial dysfunction (Fig. 9). Taken together, Maqui berry extracts may be a useful source of protective agents against blue light-induced retinal damage, especially against mitochondrial dysfunction in photoreceptors.

Many studies have been conducted on the antioxidant effects of berries containing anthocyanins [19, 20]. Here, we showed that MBE and delphinidins inhibited ROS production induced by blue light irradiation in 661W cells (Figs. 1 and 2). It was reported that the total amount of anthocyanin in MBE is 35.4% [7]. In our results, MBE was effective at protecting 661W cells from blue light-induced damage at 10 µg/mL, which corresponds to 0.71 µg/mL (0.93 µM) of D3S5G and 1.43 µg/mL (2.27 µM) of D3G5G. We demonstrated that these delphinidins exhibited their protective effects at a final concentration of 1 to 10 µM (Fig. 2). Generally, anthocyanins are known to have poor bioavailability. For example, the plasma concentration of anthocyanins ranges between 0.56 and 4.46 nmol/L following the consumption of cranberry juice (94.47 mg of anthocyanins) [21]. Absorption and metabolism of delphinidins rely on the type of sugar moiety present. Delphinidin aglycone is less bioavailable than delphinidin glycosides due to its poor aqueous solubility. The delphinidins used in this study possess glycosidic linkages at the C-3 and A-5 positions (Fig. 2B). A recent study reported that the plasma concentration of delphinidin-3-O-glucoside could reach between 21.39 and 63.55 nmol/L at 1 h after intake of MBE in healthy humans [22]. Some animal studies reported that anthocyanins are accumulated in ocular tissues [23, 24]. Further clinical studies and animal experiments are necessary to investigate the protective effects of MBE against blue light-induced retinal damage.

Vision relies on high ATP production through mitochondrial activity in photoreceptors. Blue light has been reported to decrease mitochondrial membrane potential [2]. Elevated mitochondrial ROS production is associated with impaired mitochondrial membrane potential and fragmentation [25]. Mitochondrial ROS mediates the activation of Nrf2 and induces the expression of antioxidant genes. We revealed the nuclear transport of Nrf2 and mitochondrial fragmentation at 2 h following blue light exposure (Fig. 3). It has been reported that mitochondrial complex activity is impaired by the exposure to short-wavelength light in Drosophila probably through the absorption by porphyrin [26]. Furthermore, blue light alters the mitochondrial dynamics-related protein and stimulates mitochondrial fission in retinal neuronal R28 cells, which can be attenuated by the inhibition of mitochondrial fission by Mdivi-1 [27]. In the present study, we also showed that Mdivi-1 inhibited blue-light-induced fragmentation of mitochondria in 661W cells (Fig. 5). Overall, our findings indicate that mitochondria play a crucial role in blue light-induced photoreceptor degeneration.

As the energy currency of the cell, ATP is involved in a variety of cellular events. The vacuolar ATPase (v-ATPase), an ATP-driven proton pump, is responsible for the acidification of the lysosomal lumen and is also known as a component of the mTOR pathway [28]. Blue light exposure resulted in the suppressed phosphorylation of S6RP (Fig. 3). A previous report showed that ATP depletion induced by mitochondrial inhibition causes mTORC1 inhibition [29]. It is possible that blue light-induced lysosomal dysfunction would be caused by secondary effects of the decrease of ATP production. Additionally, mitochondrial ROS also mediates lysosomal membrane permeability, and scavenging mitochondrial ROS can delay LMP [30]. In the present study, MBE and delphinidins inhibited the LMP induced by blue light irradiation. Lysosomal dysfunction has been implicated in various diseases including lysosomal storage diseases and neurodegeneration [31]. Rotenone, which has been studied in a well-established Parkinson’s disease model, increases mitochondrial ROS production by inhibiting complex I. It has been shown that an anthocyanin-rich extract suppresses the neurotoxic effects of rotenone in a primary cell culture [32]. Consistently, MBE and delphinidins ameliorated the cell death that occurred in the presence of rotenone (Fig. 9), suggesting that MBE and delphinidins would have neuroprotective effects on tissues other than the retina.

## Conclusion

The present study demonstrates that mitochondria are susceptible to blue light-induced damage in retinal photoreceptor cells. Delphinidins contained in Maqui berry extract exhibit protective effects against this cellular damage, indicating that they are useful dietary bioactive compounds for vision health.

### Electronic supplementary material

Below is the link to the electronic supplementary material.

**Supplementary Material 1:** The original images of immunoblotting. The boxed areas are presented in the indicated figures

## Data Availability

The datasets during the current study are available from the corresponding author upon reasonable request.

## Abbreviations

AMD: age-related macular degeneration
AO: Acridine Orange
ATF4: activating transcription factor 4
D3G5G: delphinidin 3,5-O-diglucoside
D3S5G: delphinidin 3-O-sambubioside-5-O-glucoside
DAPI: 4´,6-diamidino-2-phenylindole
DMEM: Dulbecco’s Modified Eagle’s Medium
ER: endoplasmic reticulum
LED: light-emitting diode
MBE: Maqui berry extract
Mdivi-1: mitochondria division inhibitor 1
Nrf2: nuclear factor erythroid-2-related factor 2
OCR: oxygen consumption rate
PI: propidium iodide
ROS: reactive oxygen species
RPE: retinal pigment epithelium
