# Supplementary figures and images for: Delphinidins from Maqui Berry (Aristotelia chilensis) ameliorate the subcellular organelle damage induced by blue light exposure in murine photoreceptor-derived cells
Source: BMC Complement Med Ther. 2024 Jan 2;24:3. doi: 10.1186/s12906-023-04322-z (PMC10759685; doi:10.1186/s12906-023-04322-z)

## Figure 3B

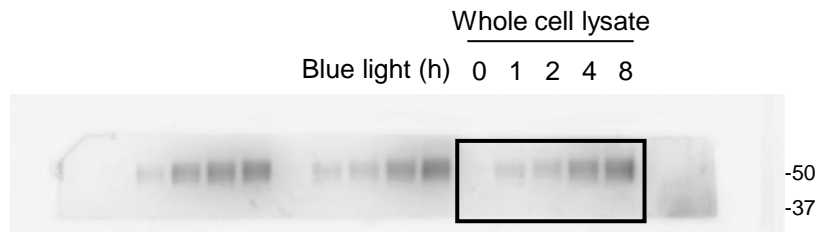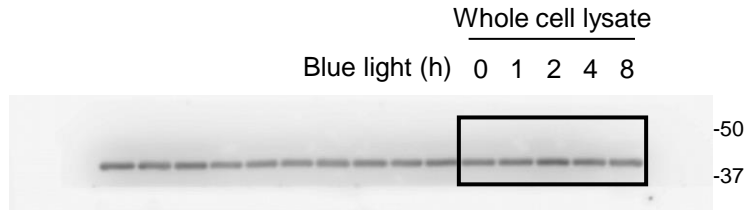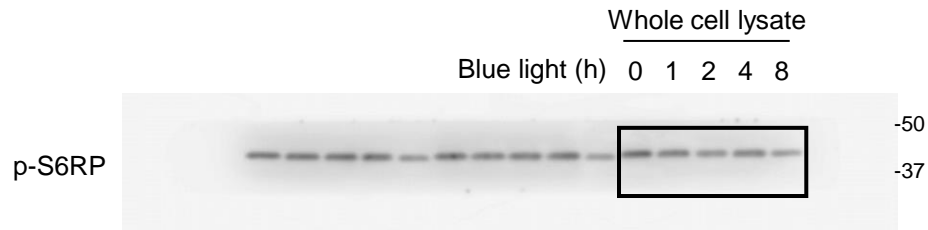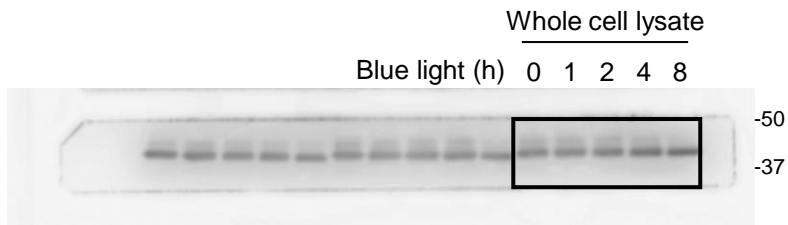

## Figure 3E

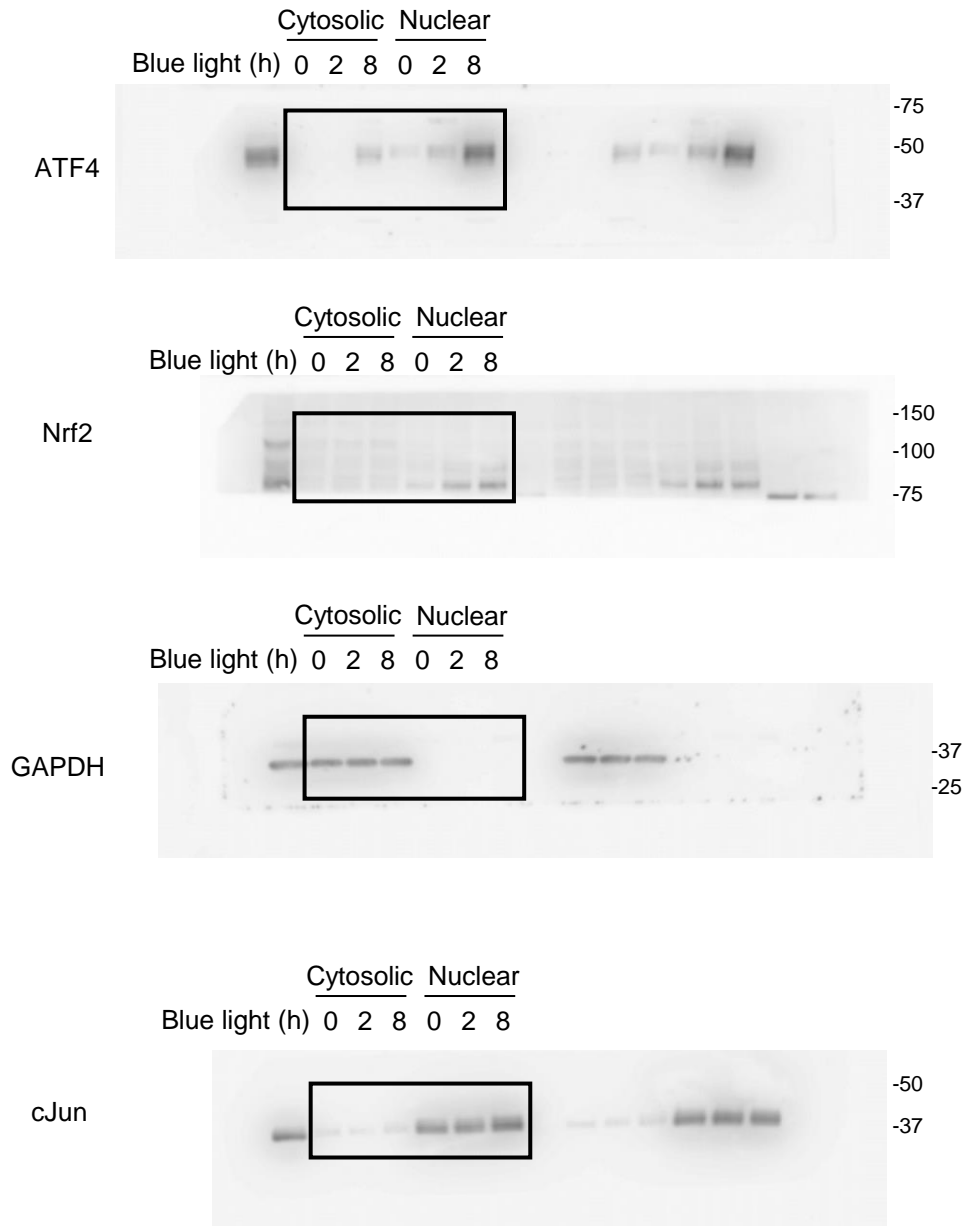

# Figure 7 A

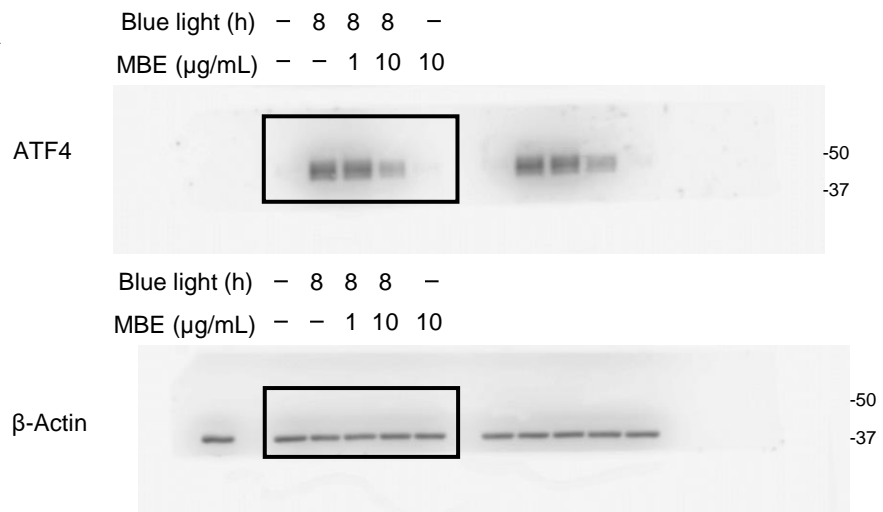

## C

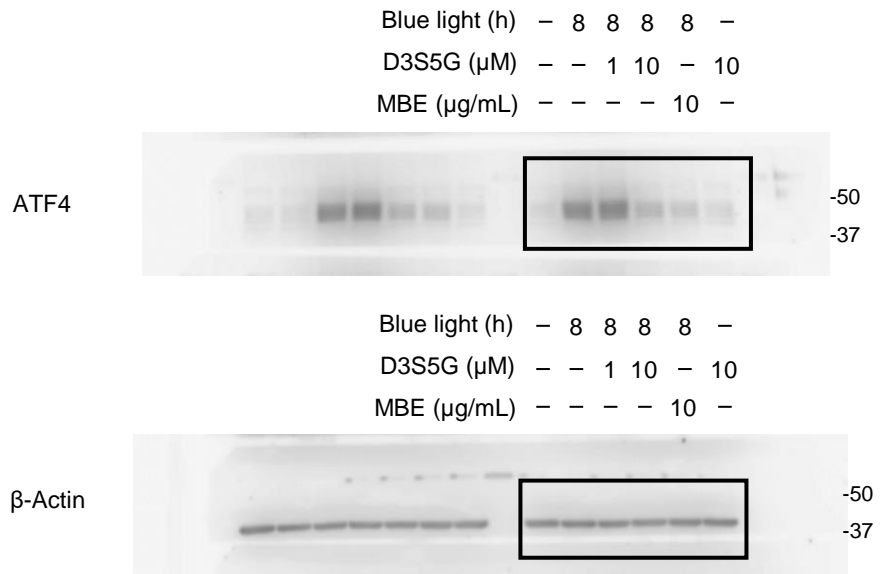

## E

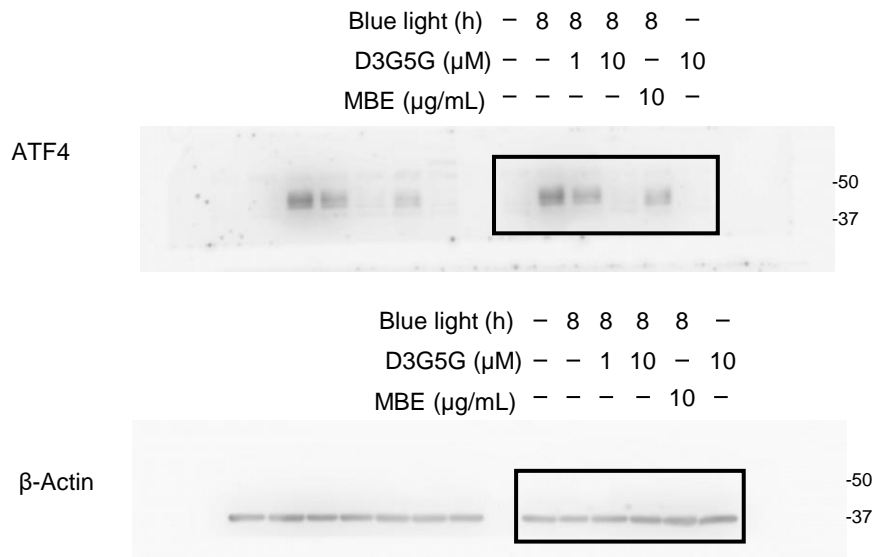

Supplement: Supplementary file 1 — Supplementary Material 1: The original images of immunoblotting. The boxed areas are presented in the indicated figures [file 12906_2023_4322_MOESM1_ESM.pdf]
